# Anticoagulant Treatment of Deep Vein Thrombosis and Pulmonary Embolism: The Present State of the Art

**DOI:** 10.3389/fcvm.2015.00030

**Published:** 2015-07-14

**Authors:** Johannes Thaler, Ingrid Pabinger, Cihan Ay

**Affiliations:** ^1^Clinical Division of Haematology and Haemostaseology, Department of Medicine I, Medical University of Vienna, Vienna, Austria

**Keywords:** venous thromboembolism, deep vein thrombosis, pulmonary embolism, anticoagulation, secondary prevention

## Abstract

Venous thromboembolism (VTE), a disease entity comprising deep vein thrombosis (DVT) and pulmonary embolism (PE), is a frequent and potentially life-threatening event. To date different agents are available for the effective treatment of acute VTE and the prevention of recurrence. For several years, the standard of care was the subcutaneous application of a low molecular weight heparin (LMWH) or fondaparinux, followed by a vitamin K antagonist (VKA). The so-called direct oral anticoagulants (DOAC) were introduced rather recently in clinical practice for the treatment of VTE. DOAC seem to have a favorable risk-benefit profile compared to VKA. Moreover, DOAC significantly simplify VTE treatment because they are administered in fixed doses and no routine monitoring is needed. Patients with objectively diagnosed DVT or PE should receive therapeutic anticoagulation for a minimum of 3 months. Whether a patient ought to receive extended treatment needs to be evaluated on an individual basis, depending mainly on risk factors determined by characteristics of the thrombotic event and patient-related factors. In specific patient groups (e.g., pregnant women, cancer patients, and elderly patients), treatment of VTE is more challenging than that in the general population and additional issues need to be considered in those patients. The aim of this review is to give an overview of the currently available treatment modalities of acute VTE and secondary prophylaxis. In particular, specific aspects regarding the initiation of VTE treatment, duration of anticoagulation, and specific patient groups will be discussed.

## Introduction

Venous thromboembolism (VTE) is the third most frequent cardiovascular disease after myocardial infarction (1, 2) and stroke (3).The estimated incidence rate of VTE is around one case per 1000 person-years (4, 5). The most frequent site of VTE is deep vein thrombosis (DVT) of the legs (6). A potentially life-threatening complication of DVT is pulmonary embolism (PE), which occurs upon embolization of a thrombus into the pulmonary arteries. The term VTE has been coined for both, DVT and PE, and will be used in this review.

For several years, the standard of care treatment of acute VTE was the subcutaneous application of low molecular weight heparin (LMWH) or fondaparinux, followed in time by the oral intake of a vitamin K antagonist (VKA) (7, 8).This regimen is highly effective for the prevention of recurrent VTE (9). However, the treatment with a VKA requires close monitoring due to a narrow therapeutic range and a relatively high rate of bleeding complications. In addition, the acute treatment of VTE requires parenteral anticoagulation with subcutaneous injections of LMWH or fondaparinux due to the delayed onset of action of VKA.

Recently a new class of agents, the so-called direct oral anticoagulants (DOAC), was introduced into clinical practice for acute and long-term treatment of VTE. Large clinical trials had shown that DOAC are effective and safe in the treatment of VTE, compared to the standard regimen with LMWH/VKA (10–13). Three DOAC, rivaroxaban, apixaban, and dabigatran, have already received approval for the treatment of VTE by the food and drug administration (FDA) and the European medicines agency (EMA). Edoxaban has been approved in the USA and Japan and is currently awaiting approval in Europe. DOAC significantly simplify the treatment of VTE because they are given in a fixed dose and no routine monitoring is needed. Moreover, in meta-analyses DOAC were associated with a significantly lower risk of bleeding complications (14, 15).

In this review, we give an overview of the present state-of-the-art for the treatment of DVT and PE. Furthermore, we mean to provide guidance for clinical decision-making with regard to the various available treatment modalities for specific patient groups and their very particular requirements.

## Considerations before Initiation of Treatment

### Hemodynamically unstable pulmonary embolism

Patients with suspected PE who are hemodynamically unstable and present with shock or hypotension are at high risk of short-term mortality (16). If PE is confirmed, such patients should be considered for thrombolysis, and in exceptional cases for surgical or catheter embolectomy (e.g., when they are not at high risk of bleeding) (16, 17). Moreover, in patients with hypotension or shock unfractioned heparin (UFH) should be used for initial anticoagulation instead of LMWH, fondaparinux or a DOAC according to the current guidelines of the European society of cardiology (ESC) (18).

The pulmonary embolism severity index (PESI) score and its simplified version can be used for discriminating between patients who need to be hospitalized or could potentially be treated in the ambulatory setting (19–22).

### High bleeding risk

According to the current American college of chest physicians (ACCP) guidelines, in patients with acute proximal DVT or PE an inferior vena cava filter might be placed, if anticoagulation is not possible due to an exceedingly high bleeding risk (23). However, the ACCP guidelines do not go into more detail and “bleeding risk” is not clearly defined. Estimation of the bleeding risk can be guided by a prospectively validated clinical score, such as the RIETE score (24) and the HEMORR2HAGES score (25). The usefulness and applicability of these scores for routine clinical practice yet remains to be established.

Regarding permanent vena cava filters it is important to consider that they are associated with a number of long-term complications like filter thrombosis and filter migration. Temporary filters must be removed within a few days, while retrievable filters can be left in place for longer periods (25).

### Impaired renal function

Low molecular weight heparin and fondaparinux are mainly eliminated by the kidneys while UFH is mainly eliminated by the reticuloendothelial system and VKA by CYP2C9 and the vitamin K epoxide reductase of the liver (26). In patients with a creatinine clearance of <30 mL/min, caution is indicated because LMWH and particularly fondaparinux are likely to accumulate, thus eventually resulting in over-anticoagulation. LMWH and fondaparinux can be monitored by measuring the peak anti-factor Xa activity (4 h after administration). However, therapeutic ranges are not clearly defined. For LMWH with a twice-daily injection schedule, a peak anti-factor Xa activity of 0.6–1.0 IU/mL is currently suggested as effective therapeutic anticoagulation (27, 28). The therapeutic range for once daily dosing is less clear and suggested to be 1.0–2.0 IU/mL (28).

Renal function is also an important factor, if anticoagulation with a DOAC is considered. In the Hokusai-VTE trial that investigated edoxaban, a reduced dose (30 mg instead of 60 mg once daily) was given to patients with a glomerular filtration rate (GFR) between 30 and 50 mg/dL, which was shown to be safe, and those with a GFR below 30 mg/dL were excluded (11). In the large controlled trials that have led to the approval of rivaroxaban, dabigatran, and apixaban, patients with a GFR below 30 mg/dL (rivaroxaban and dabigatran) or with a GFR below 25 mg/dL (apixaban) were excluded (10, 29–31). For rivaroxaban a dose reduction rule is given in the package insert for patients with a GFR between 15 and 30 mg/dL. However, we do not recommend the use of any DOAC in patients with a GFR below 30 mg/dL (or below 25 mg/dL for apixaban) because of the lack of clinical data.

## Treatment Modalities for Acute Deep Vein Thrombosis and Pulmonary Embolism

**Figure 1:**
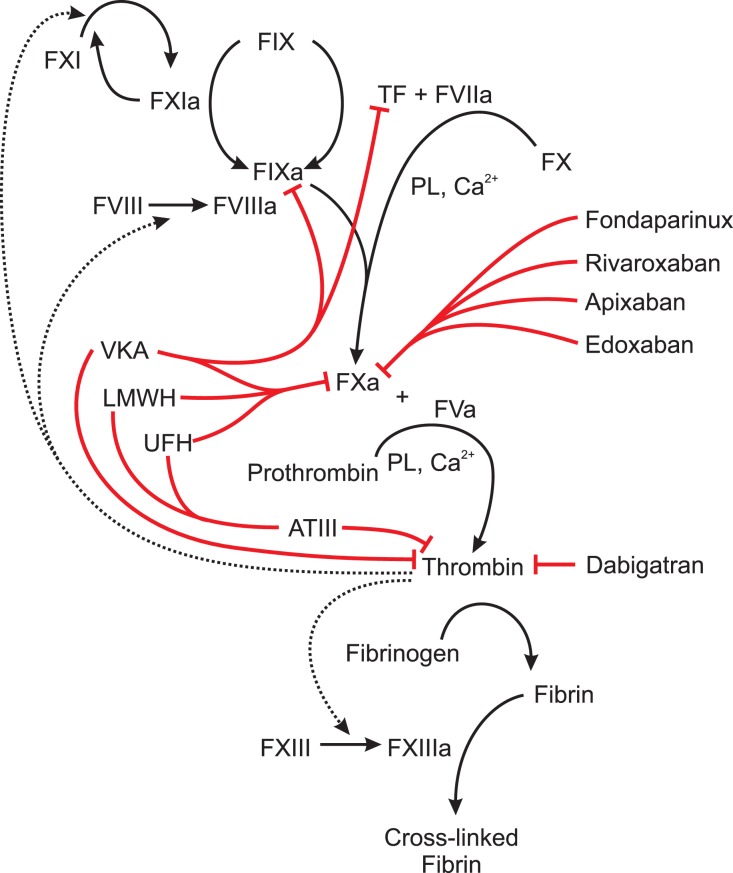
**Schematic overview of the inhibitory effects of different anticoagulants in the blood coagulation cascade**. Abbreviations: AT III, antithrombin III; LMWH, low molecular weight heparin, PL, phospholipid; TF, tissue factor; VKA, vitamin K antagonists, UFH, unfractioned heparin; F###: blood coagulation factor denoted in Roman numerals. Active forms are denoted by a small “a” added to the Roman number, red lines: inhibition by anticoagulants, dotted lines: positive feedback loops by thrombin. VKA inhibit the synthesis of the coagulation factors II, VII, IX, and X by acting as a competitive inhibitor of the enzyme vitamin K epoxide reductase. LMWH and UFH bind to AT thereby increasing the ability of ATIII to inhibit thrombin up to 1000-fold. LMWH and UFH also directly inhibit FXa. UFH rather target AT III and LMWH rather target FXa. Fondaparinux, rivaroxaban, apixaban, and edoxaban directly inhibit FXa. Dabigatran directly inhibits thrombin.

A summary of the design and characteristics of the studies is presented in Table 1. A schematic overview of the inhibitory effects of different anticoagulants in the coagulation cascade is given in Figure 1. For detailed information on contraindications and drug interactions, the reader is referred to the package insert/prescribing information of the respective agent.

**Table 1:** **Characteristics of the studies that investigated the efficacy of DOAC compared to VKA for treatment of VTE**.

Study	Einstein DVT + PE	AMPLIFY	RE-COVER I + II	Hokusai-VTE
Drug	Rivaroxaban	Apixaban	Dabigatran	Edoxaban

Total number of patients	8246	5365	5107	8240

Target	Factor Xa-inhibition	Factor Xa-inhibition	Thrombin-inhibition	Factor Xa-inhibition

Primary efficacy outcome	Recurrent venous thromboembolism			

Principal safety outcome	Major- or clinically relevant non-major bleeding			

Study design	Open-label, randomized non-inferiority trial	Randomized, double-blind trial	Randomized, double-blind, non-inferiority trial	Randomized, double-blind, non-inferiority trial

Time INR in therapeutic range^a^	57.7%	61%	60%	63.5%

Regimen/dose (mg)	15 mg twice daily for the first 3 weeks followed by 20 mg once daily	10 mg twice daily for the first 7 days, followed by 5 mg twice daily	Initial treatment (at least 5 days) with parenteral anticoagulant, followed by 150 mg twice daily	Initial treatment (at least 5 days) with parenteral anticoagulant, followed by 60 mg once daily

Treatment duration	3-, 6-, and 12 months	6 months	6 months	3–12 months duration determined by the treating physician based on patient’s clinical features and preference

Dose adjustment	No	No	No	Yes^b^

Dose reduction criteria	Not assessed	Not assessed	Not assessed	30 mg once daily in patients with a CrCl 30–50 mL/min, body weight ≤60 kg or concomitant treatment with potent P-glycoprotein inhibitor

Most important exclusion criteria as listed in the publications (full list of exclusion criteria provided in the study protocols)	Another indication for VKA, CrCl <30 mL/min, liver disease, bacterial endocarditis, contraindications for anticoagulant treatment, systolic blood pressure >180 mmHg or diastolic blood pressure >110, childbearing potential without proper contraceptive measures, pregnancy or breast feeding, concomitant use of strong cytochrome P450 3A4 inhibitors, bacterial endocarditis	Contraindications to heparin or warfarin, CrCl <25 mL/min or creatinine level >2.5 mg/dL, liver disease, cancer with long-term treatment with LMWH, provoked DVT or PE in the absence of a persistent risk factor for recurrence, another indication for long-term anticoagulation therapy, dual antiplatelet therapy, aspirin at a dose of more than 165 mg daily, hemoglobin <9 mg/dL, platelet count <100.000/mm^3^	Another indication for a VKA or heparin, CrCl <30 mL/min, liver disease, PE with hemodynamic instability or requiring thrombolytic therapy, recent unstable cardiovascular disease, high risk of bleeding, liver disease, contraindication to heparin, pregnancy or risk of becoming pregnant long-term antiplatelet therapy (aspirin ≤100 mg accepted), life expectancy less than 6 months	Another indication for VKA, CrCl <30 mL/min, contraindications to heparin or warfarin, cancer with long-term treatment with LMWH, treatment with aspirin at a dose of more than 100 mg daily or dual antiplatelet therapy

*^a^Target INR (vitamin K antagonist) in all studies: 2.0–3.0*.

*CrCL, creatinine clearance; VKA, vitamin K antagonist; LMWH, low molecular weight heparin; DVT, deep vein thrombosis; PE, pulmonary embolism*.

*^b^In the Hokusai-VTE study, 17.8% of patients received the adjusted dose of edoxaban (30 mg once daily) at randomization*.

### LMWH or fondaparinux followed by VKA

The standard treatment of acute DVT and PE for many years has been the initial administration of a LMWH or fondaparinux at a therapeutic dose, followed by the (initially overlapping) oral intake of a VKA. The parenteral drug can be stopped when an international normalized ratio (INR) of 2.0 is reached on two successive days. Parenteral anticoagulation should be performed for at least 5 days.

Vitamin K antagonists are highly effective in the prevention of recurrent VTE with a relative risk reduction of ~85% compared to placebo (9). However, it is a drawback that the intake of VKA needs to be closely monitored due to the narrow therapeutic range and that even in specialized centers only about 60% of the INR values are within the therapeutic range (32) (Table 1). Moreover, a relatively high risk of intracranial bleeding (1.1 per 100 patient-years) and a case-fatality rate of major bleeding of 13.4% were reported in a meta-analysis by Linkins et al. (30).

### Direct oral anticoagulants

Rivaroxaban is a direct factor Xa inhibitor and the first DOAC that was approved in 2012 for the treatment of VTE. The initial dose of rivaroxaban for the treatment of acute VTE is 15 mg twice daily for 3 weeks followed by 20 mg once daily for at least 3 months. This fixed dose treatment regimen was chosen for all included patients irrespective of their body weight or age. The efficacy of rivaroxaban in the treatment of acute DVT and PE was demonstrated in two large open-label trials (Einstein DVT and Einstein PE) (30, 31). These studies compared rivaroxaban with the standard of care treatment (LMWH followed by VKA) and were designed as non-inferiority studies. In both studies rivaroxaban proved as effective as LMWH followed by VKA with similar bleeding rates. In the Einstein-Extension study (published together with the Einstein PE study), the extended anticoagulant treatment for 12 months with 20 mg rivaroxaban once daily was tested in comparison to placebo. Rivaroxaban substantially reduced the rate of long-term VTE at the cost of a moderately increased risk of bleeding.

Apixaban, also a direct factor Xa inhibitor, was approved in 2014 for the treatment of VTE. The initial dose of apixaban is 10 mg twice daily for 7 days followed by apixaban 5 mg twice daily for at least 3 months. Apixaban was tested in the AMPLIFY study, a double-dummy double-blind trail, and was non-inferior compared to standard treatment (12). Major bleeding occurred less frequently under the treatment with apixaban. In the AMPLIFY Extension study patients were randomized into two different doses of apixaban (5 or 2.5 mg twice daily) or placebo for testing VTE recurrence during 12 months of extended treatment. Both treatment doses of apixaban similarly reduced the risk of recurrent VTE without an increased risk of major bleeding (29). Based on these findings, patients designated for infinite anticoagulation should be given apixaban 5 mg twice daily for 6 months followed by apixaban 2.5 mg twice daily.

Dabigatran is a direct thrombin inhibitor that was also approved in 2014 for the treatment of VTE. Two double-blind, double-dummy studies (RE-COVER and RE-COVER II) found that Dabigatran was non-inferior compared to standard treatment and no differences with regard to major bleeding episodes were found. Initial treatment is started with a LMWH at a therapeutic dose for 5–7 days followed by dabigatran 150 mg twice daily for at least 3 months. According to the prescribing information individuals older than 79 years should get dabigatran at a reduced dose. However, this recommendation is not based on clinical data, as no dose adjustment was performed in the RE-COVER studies.

The direct factor Xa inhibitor edoxaban has been approved in the USA and Japan and is currently under regulatory review in Europe. Edoxaban was investigated in the Hokusai-VTE study, a double-blind, double-dummy study that compared edoxaban to standard treatment (11). Patients initially received LMWH for 5 days followed by edoxaban 60 mg once daily, which was reduced to edoxaban 30 mg once daily in patients with a creatinine clearance of 30–50 mL/min or a body weight below 60 kg. Edoxaban was administered for a flexible duration of 3–12 months, and a 12-month follow-up was performed in all patients. Edoxaban was non-inferior compared to standard treatment and less clinically relevant non-major bleedings occurred, which was the main safety outcome. In a sub-group analysis, a favorable efficacy outcome was noted in patients with severe PE who presented with evidence of right ventricular dysfunction (defined as NT pro-BNP ≥500 pg/mL) in the edoxaban-treatment arm compared to warfarin.

## Duration of Anticoagulation

Venous thromboembolism is a disease that may often recur, with a 5-year recurrence risk of up to 20–25%. Extended thromboprophylaxis is effective in preventing recurrence of VTE, but it is also associated with a substantially increased risk of major bleeding. Whether a patient should receive extended thromboprophylaxis thus needs to be evaluated on an individual basis, mainly depending on risk factors determined by characteristics of the thrombotic event and by patient-related factors.

The ACCP in its most current guidelines from 2012 recommends anticoagulation for a minimum of 3 months for all patients with DVT or PE irrespective of the underlying cause (33). According to the ACCP guidelines anticoagulation should be stopped after 3 months in patients with a transient risk factor like surgery, trauma, immobilization, pregnancy, or female hormone intake (34–37). The ACCP guidelines do not address treatment with DOAC. In the Amplify study investigating Apixaban for the treatment of acute VTE patients with a transient risk factor and “less than 6 months of anticoagulant treatment planned” were excluded (13). Therefore, the anticoagulation with Apixaban might not be recommendable for such patients because of a lack of data. The other large clinical studies investigating dabigatran, rivaroxaban, and edoxaban for the treatment of VTE included patients with a transient risk factor for VTE, but the optimal duration of anticoagulation was not specifically investigated for this particular patient group and therefore remains to be elucidated. Whether recommendations for the duration of anticoagulation will be influenced by the designs of the DOAC trials still has to be awaited until the publication of the revised guidelines.

Infinite anticoagulation should be considered in patients with a spontaneous proximal DVT (from the vena poplitea upwards) or PE, as they are at high risk of recurrence. The benefits of anticoagulation have to be weighed against the risk of bleeding and personal preferences. Therefore, an individualized treatment approach should be pursued. In line with this approach, a risk assessment model was published for the identification of patients with unprovoked VTE in whom a only limited duration of anticoagulation can be considered as relatively safe (38).

Heritable thrombophilic defects are found in at least one-third of patients with acute VTE (39). Screening for heritable thrombophilia may therefore be promising and has been advocated. However, in unselected patients with a first episode of VTE it has been shown that testing for heritable thrombophilia does not allow the prediction of VTE recurrence (40–45). Moreover, it has to be considered that VTE is a multifactorial disease and that a negative finding from thrombophilia testing could result in a false sense of safety in a patient or even the treating physician, as a third of patients with recurrent VTE do not have a thrombophilic defect (46). We conclude that thrombophilia screening should not be performed on a routine basis. In specific cases, such as younger patients with VTE (including oral contraceptive users), investigation of lupus anticoagulants, antiphospholipid antibodies, antithrombin III deficiency, and protein C and protein S deficiency might be performed on a case-by-case basis.

## Treatment of VTE in Specific Patient Groups

### Cancer patients

The presence of malignancy is a strong and independent risk factor for the occurrence of VTE (47). The reported incidence rates of VTE in cancer patients vary widely, and strongly depend on several patient-, treatment-, and cancer-related risk factors (48). The occurrence of VTE has a dismal impact on the course of malignancy and adds to the morbidity and mortality of cancer patients (49).

The treatment of VTE is more challenging in cancer patients than in the non-cancer population, as cancer patients are more likely to develop recurrent VTE during anticoagulation and are also at increased risk of bleeding (50).

In the 2013 clinical practice guideline update the American Society of Clinical Oncology (ASCO) recommended anticoagulation with a LMWH for at least six months after VTE in cancer patients over treatment with a VKA or UFH (51, 52). This recommendation is based on three clinical trials that found superiority of LMWH over VKA for the prevention of recurrent cancer-related VTE (53–55). Extended thromboprophylaxis (beyond 6 months) should be considered for patients with active malignancy and particularly for those with metastatic disease and/or ongoing chemotherapy. The decision to continue anticoagulation in cancer patients should be reassessed in regular intervals considering the risk of bleeding, quality of life, life expectancy, and patient preference.

A recent meta-analysis including five randomized controlled trials found that DOAC are at least comparable to VKA in the treatment of cancer-related VTE (56). However, interventional trials comparing the efficacy of DOAC with the standard of care treatment in cancer patients, i.e., LMWH, are missing so far. A randomized controlled trial comparing edoxaban to dalteparin in the treatment of cancer-related VTE is currently starting recruitment (2015, March 6, retrieved from https://clinicaltrials.gov/ct2/show/NCT02073682?term=edoxabancancer&rank=1).

### Pregnant women

Pregnancy is associated with a two-fold increased risk of developing VTE (57). Fatal PE is the most common cause of death in pregnant women in Western countries (58).

Low molecular weight heparins are the treatment of choice for pregnant women with acute VTE because LMWHs do not cross the placenta and have already been used in a large number of patients. According to the current ACCP guidelines weight-adjusted therapeutic doses of LMWH should be used for the treatment of VTE during pregnancy and should be continued for at least 6 weeks following delivery (for a minimum of 3 months of therapy). Twenty-four hours prior to planned delivery discontinuation of LMWH is recommended (33).

In contrast, VKA must not be given to pregnant women because they cross the placenta and intake of a VKA is associated with embryopathy, particularly in the first trimester (59). DOAC have not been investigated in pregnant women so far and are therefore contraindicated (60).

### Elderly patients

In elderly patients the treatment of VTE is challenging. Specific age-related problems are (amongst others) decreased kidney function, decreasing body weight, dementia, co-morbidities, and an increased tendency to fall over. Moreover, elderly patients were often excluded from clinical trials and therefore some data from clinical trials might not be applicable to these patients.

Age is an independent risk factor for VTE and a high prevalence of asymptomatic DVT events was found in patients above 80 years (61). Also the risk of bleeding complications is increased (60). However, the rate of fatal PE was 2.5-times higher than the rate of fatal bleeding in patients below 80 years and 4.5-times higher in those older than 80 years. Thus, the risk of fatal PE appears to be more alarming than the bleeding risk.

All treatment regimens for VTE, except for dabigatran, are similar for elderly and younger patients. Only dabigatran should be taken at a reduced dose (110 mg twice daily) in patients aged 80 years or older. As mentioned, this regimen has not been tested in clinical trials.

Specific VTE treatment regimens for elderly patients still remain to be tested in future investigations. Sub-group analyses of the large clinical trials that compared DOAC to VKA indicate that DOAC have a better risk-benefit profile. A meta-analysis comparing DOAC to VKA found significantly fewer recurrent VTE events and a significant reduction in major bleeding in elderly patients treated with a DOAC (14). In a pooled analysis of the two EINSTEIN trials significantly less bleeding was found in fragile patients (defined as age above 75 years or creatinine clearance below 50 mL/min, or body weight below 50 kg) taking rivaroxaban compared to those taking a VKA (62).

## Conclusion

Venous thromboembolism is a frequent and potentially life-threatening event. To date different agents are available for the effective treatment of acute VTE and the prevention of recurrence. DOAC seem to have a more favorable risk-benefit profile compared to VKA.

Based on individual patient characteristics and laboratory parameters, patient-specific treatment modalities should be tailored and clinical decision-making should be guided by current guidelines, risk assessment scores, and data from randomized controlled trials. Special attention has to be paid to the question whether extended anticoagulation for secondary VTE prophylaxis is indicated.

In specific patient groups like pregnant women, cancer patients, and elderly patients, treatment of VTE is more challenging than that in the general population. Several additional considerations have to be taken into account in such patients and treatment regimens should be determined by experts.

## Conflict of Interest Statement

The authors declare that the research was conducted in the absence of any commercial or financial relationships that could be construed as a potential conflict of interest.
